# Coproantigen ELISA Effectively Detects Moderate-To-High *Fasciola* Egg Counts with Limited Sensitivity at Low Levels in Field Settings

**DOI:** 10.21315/tlsr2026.37.1.11

**Published:** 2026-03-31

**Authors:** Naim Che-Kamaruddin, Lokman Hakim Idris, Nur Mahiza Md Isa

**Affiliations:** 1 Tropical Infectious Diseases Research and Education Centre (TIDREC), Higher Center of Excellence (HICoE), Universiti Malaya, 50603 Kuala Lumpur, Malaysia; 2 Department of Veterinary Pre-Clinical Science, Faculty of Veterinary Medicine, Universiti Putra Malaysia, 43400 Serdang, Selangor, Malaysia; 3 Department of Pathology and Microbiology, Faculty of Veterinary Medicine, Universiti Putra Malaysia, 43400 Serdang, Selangor, Malaysia

**Keywords:** Coproantigen ELISA, Farm, Liver Fluke, Parasite Egg Count, Koproantigen ELISA, Ladang, Cacing Hati, Kiraan Telur Parasit dalam Najis

## Abstract

Detection of *Fasciola* eggs in ruminant faecal samples typically occurs around 15 weeks post-infection during patent infections. The recent introduction of coproantigen ELISA (cELISA) enables rapid antemortem diagnosis of fasciolosis, highlighting the need to evaluate its diagnostic performance under the field conditions. This study aimed to assess the usability and effectiveness of a cELISA kit for detecting *Fasciola gigantica* infections in free-grazing cattle from an endemic area, and to explore the relationship between cELISA results and conventional faecal egg counts obtained via the Flukefinder® sedimentation method. A total of 92 faecal samples (46 positive and 46 negative by faecal egg count) were analysed. The cELISA detection limit was identified at 4.5 eggs per gram (epg), achieving 100% positivity above this threshold. A moderate, statistically significant positive correlation was observed between faecal egg counts and cELISA optical density (OD) values (Spearman’s *r* = 0.716, *p* < 0.01). Additionally, an odds ratio of 1.96 (*p* < 0.01) indicated that the likelihood of higher coproantigen levels nearly doubles with each additional egg detected. These findings suggest that cELISA is a sensitive and practical tool for diagnosing active *Fasciola* infections with moderate to high egg burdens, complementing traditional faecal sedimentation tests. Together, these approaches improve field diagnostics, enable targeted strategies and support effective fasciolosis control in endemic settings.

## Abstrak

Pengesanan telur Fasciola dalam sampel najis ruminan lazimnya berlaku sekitar 15 minggu selepas jangkitan semasa fasa jangkitan paten. Pengenalan terkini kaedah ELISA koproantigen (coproantigen ELISA, cELISA) membolehkan diagnosis fasciolosis secara antemortem dengan lebih cepat, sekaligus menekankan keperluan untuk menilai prestasi diagnostiknya dalam keadaan lapangan sebenar. Kajian ini bertujuan menilai kebolehgunaan dan keberkesanan kit cELISA dalam mengesan jangkitan Fasciola gigantica dalam lembu yang meragut bebas di kawasan endemik, serta meneroka hubungan antara keputusan cELISA dengan kiraan telur dalam najis yang diperoleh melalui kaedah pemendapan Flukefinder®. Sebanyak 92 sampel najis (46 positif dan 46 negatif berdasarkan kiraan telur dalam najis) telah dianalisis. Had pengesanan cELISA dikenal pasti pada 4.5 telur per gram (epg), dengan kadar pengesanan 100% positif bagi sampel yang melebihi bilangan telur tersebut. Analisis korelasi menunjukkan hubungan positif sederhana yang signifikan secara statistik antara kiraan telur najis dan nilai ketumpatan optik (OD) cELISA (Spearman r = 0.716, p < 0.01). Selain itu, nisbah ganjil sebanyak 1.96 (p < 0.01) menunjukkan bahawa kebarangkalian peningkatan tahap koproantigen hampir berganda bagi setiap pertambahan satu telur yang dikesan. Dapatan ini mencadangkan bahawa cELISA merupakan kaedah yang sensitif dan praktikal untuk mendiagnosis jangkitan Fasciola aktif dengan beban telur sederhana hingga tinggi, serta dapat melengkapkan ujian pemendapan najis konvensional. Gabungan kedua-dua pendekatan ini berpotensi meningkatkan ketepatan diagnostik di lapangan, membolehkan strategi rawatan yang lebih bersasar, serta menyokong kawalan fasciolosis yang lebih berkesan di kawasan endemik.

**HIGHLIGHTS**

- 100% coproantigen ELISA (cELISA) positivity was achieved only at ≥ 4.5 egg per gram (epg), indicating the assay’s reliable performance at moderate-to-high *Fasciola* infection intensities.
- Odds ratio analysis showed that for each additional *Fasciola* egg detected, the odds of cELISA positivity nearly doubled (OR = 1.96, *p* < 0.01).
- A moderate positive correlation was observed between *Fasciola* faecal egg count (FFEC) and coproantigen levels (Spearman *r* = 0.716, *p* < 0.01).

## INTRODUCTION

Fasciolosis is an emerging neglected tropical disease that has a significant detrimental impact on ruminant livestock production (Mezo *et al*. 2004; Alvarez-Rojas *et al*. 2014; Alba *et al*. 2021). Fasciolosis is typically diagnosed coproscopically through faecal sedimentation to detect the *Fasciola* eggs. However, the *Fasciola* eggs are detectable during patent infection period which starts 15 weeks after ingesting infectious *Fasciola* metacercariae (Mezo *et al*. 2004). Other diagnostic includes serological assays of host IgG antibody response, which is less accurate for active infection diagnosis as the IgG remains detectable for months even after parasitic clearance. Abattoir examinations detect infections post-mortem, limiting early detection to after slaughter. This delay allows *Fasciola* to persist and spread within the herd before preventive measures can be implemented (Mazeri *et al*. 2017). However, the abattoir examinations still provide crucial insights into the extent of infection, tissue damage and other pathological changes (Alvarez-Rojas *et al*. 2014).

Coproantigen enzyme-linked immunosorbent assay (cELISA) has emerged over the past decade as a diagnostic for fasciolosis detection in ruminants. This cELISA captures metabolic antigens produced by newly excysted juvenile (NEJ) and adult *Fasciola* (Mezo *et al*. 2004; Kajugu *et al*. 2015), demonstrating high sensitivity and specificity for fasciolosis detection in sheep and cattle (Kajugu *et al*. 2015). The primary antigen secreted by *Fasciola* is cathepsins-L, which interacts with the host’s digestive enzymes and acids, circulating and retaining in the biliary system (Mezo *et al*. 2004; Kajugu *et al*. 2015). Additionally, cathepsin-L can be detected even during the pre-patent period even with a low fluke burden, as low as two flukes (Mezo *et al*. 2004; Martínez-Sernández *et al*. 2018).

Fasciolosis surveillance utilising cELISA is widely employed to detect *Fasciola hepatica* in temperate regions (Valero *et al*. 2009; Gordon *et al*. 2012; Brockwell *et al*. 2013; Kajugu *et al*. 2015). However, its application and validation for *F. gigantica* in tropical regions, such as Malaysia, remain comparatively limited and understudied despite the high burden of *F. gigantica* in these areas (Valero *et al*. 2009). This lack of region-specific evaluation represents a critical research gap complexified by ecological, epidemiological and host factors distinct to tropical environments. The sensitivity of cELISA for both *Fasciola* species is comparable, detected about four to seven weeks before eggs appear in sheep (six to nine weeks for *F. hepatica* and seven to 11 weeks post-infection (WPI) for *F. gigantica*) (Valero *et al*. 2009). During patent infection, eggs are released into the environment through defaecation, perpetuating fasciolosis. Therefore, early and accurate detection is essential for controlling the spread and minimising economic losses (Bishop 2012; Mazeri *et al*. 2017).

Given the limited data on the effectiveness of cELISA for diagnosing *F. gigantica* infections under tropical field conditions, the present study aimed to compare the sensitivity of faecal sedimentation using Flukefinder® with that of cELISA for diagnosing fasciolosis in free-grazing cattle in an area endemic to *F. gigantica*. This comparison was undertaken to evaluate the usability and diagnostic performance of cELISA under field conditions and to explore how it may complement traditional egg counts to improve the diagnosis of fasciolosis.

## MATERIALS AND METHODS

### Ethical Approval

Ethical approval for this study was obtained from Universiti Putra Malaysia (UPM) [Clearance No. UPM/IACUC/AUP007/2019]. The work involved non-experimental animals, and only faecal samples were collected. Sampling was entirely non-invasive and non-painful, ensuring no harm or distress to the animals.

### Faecal Sample and Flukefinder® Sedimentation Egg Count Test

Sample collection details are outlined in our earlier study (Che-Kamaruddin *et al*. 2024). Briefly, ruminant faecal samples were collected for fasciolosis surveillance on farms in Taiping, Malaysia, from February to August 2020. For the present study, the analysis focused exclusively on cattle samples, as the cELISA kit used was optimised for faecal samples from sheep and cattle. From the larger pool of cattle faecal samples collected during surveillance, 92 samples were selected using simple random sampling. Each was assigned a number, and random numbers were generated using Microsoft Excel’s random number function to ensure unbiased selection. These samples were then subjected to Flukefinder® sedimentation for *Fasciola* faecal egg count (FFEC). Based on the sedimentation results, samples were subsequently categorised into two study groups: FFEC+ve (*n* = 46; *Fasciola* eggs detected) and FFEC-ve (*n* = 46; no *Fasciola* eggs detected).

The FFEC was performed using a modified Flukefinder® sedimentation method (Richard Dixon, ID, USA). Flukefinder® is more sensitive than conventional sedimentation, with a 100% recovery rate for *Fasciola* eggs (Kahl *et al*. 2023). Briefly, 2 g of faecal sample was mixed with 30 mL of treated water and poured into the Flukefinder®. The filtrate was allowed to sediment in a 5 cm vial for 2 min, after which the supernatant was discarded. The sediment was transferred to a gridded Petri dish, stained with three drops of 10% methylene blue, and examined under a stereomicroscope at 25**×** to 400**×** magnification for egg counting. The number of eggs observed was expressed as eggs per gram (epg) of faeces. In the present study, infection intensity thresholds based on epg were not applied. Instead, individual egg counts were recorded and directly analysed against cELISA optical density (OD) values to assess the relationship between egg detection and coproantigen levels in faecal samples.

*Fasciola* eggs were identified based on shape, colour and operculum morphology, following Gordon *et al*. (2012). Specifically, *Fasciola* eggs were characterised by their golden-yellow to brownish shell, elongated-ellipsoidal shape (approximately 130 μm–150 μm in length and 60 μm– 90 μm in width), and a prominent operculum with a distinct “shoulder spine” at one end. These features differentiate *Fasciola* eggs from those of other trematodes, such as *Paramphistomum*, which are typically smaller (100 μm–120 μm), lack a prominent operculum, and have a more translucent appearance with less defined internal structures (Gordon *et al*. 2012; Taylor *et al*. 2016). To ensure accurate identification, samples were cross-checked under light microscopy at 100× and 400× magnification by trained parasitologists. Multiple eggs per positive sample were assessed to confirm consistency in size, shape, colour and operculum morphology, reducing the likelihood of misidentification with morphologically similar trematode eggs.

### Preparation of Faecal Supernatant for cELISA

Faecal supernatants were prepared using a 1:1 ratio of faecal samples to ProClin® 300 (Sigma-Aldrich, CAS#55965-84-9), with 2 g of each cattle faecal sample. Samples were vortexed to ensure homogeneity and prevent clumping, then incubated overnight at 4°C. After incubation, the suspension was thawed to room temperature, vortexed and centrifuged at 1,000 x g for 10 min to obtain the supernatant. A 500 μL aliquot of each supernatant was stored at −20°C. The supernatant remains viable for *Fasciola* coproantigen detection for up to six weeks (Gordon *et al*. 2012).

### cELISA Analysis

In this study, a commercial semi-quantitative cELISA kit (MM3-COPRO-BIOK 201, Bio-X Diagnostics) was used following the manufacturer’s instructions. The kit features an indirect sandwich cELISA design with monoclonal and polyclonal antibodies on alternate strips to reduce false positives. It includes an avidin-peroxidase conjugate for sensitive *Fasciola* coproantigen detection and an MM3 monoclonal antibody, noted for high sensitivity and specificity of detecting even one NEJ or adult *Fasciola* (Martínez-Sernández *et al*. 2016). The second conjugate was the MM3 monoclonal antibody (mAb), which is the most sensitive and specific mAbs for binding with *Fasciola* coproantigen (Mezo *et al*. 2004).

For the assay, 100 μL of each supernatant was added to wells coated with monoclonal and polyclonal antibodies, with duplicates for averaging. Reference *Fasciola* antigen was used as a positive control, and deionised distilled water served as a blank control to measure nonspecific binding. Optical densities (OD) were read at 450 nm, with a cut-off value of 8 determined from the QC datasheet; OD values above this threshold were considered positive.

### Statistical Analysis

The Kolmogorov-Smirnov test was used to examine the data distribution. One-way ANOVA was used to analyse the OD results, assessing total variance and coefficient of variation (CV) for each sample, expressed as a percentage. Spearman’s rank correlation and linear regression were used to evaluate the correlation between OD values from cELISA and FFEC, with significance set at *p* < 0.01. All statistical analyses were conducted using R statistical software (version 1.3.1073).

## RESULTS

In the present study, the cELISA OD readings for blank controls and polyclonal antibody-coated wells were consistently below the cut-off value, while positive controls with crude *Fasciola* antigen exceeded the cut-off, validating the OD results. The *Fasciola* egg counts in the FFEC+ve samples ranged from 1 egg per 2 grams (0.5 epg) to 113 eggs per 2 grams (56.5 epg), with a median value of 2 epg, indicating a skewed distribution in the free-grazing cattle samples.

**FIGURE 1:**
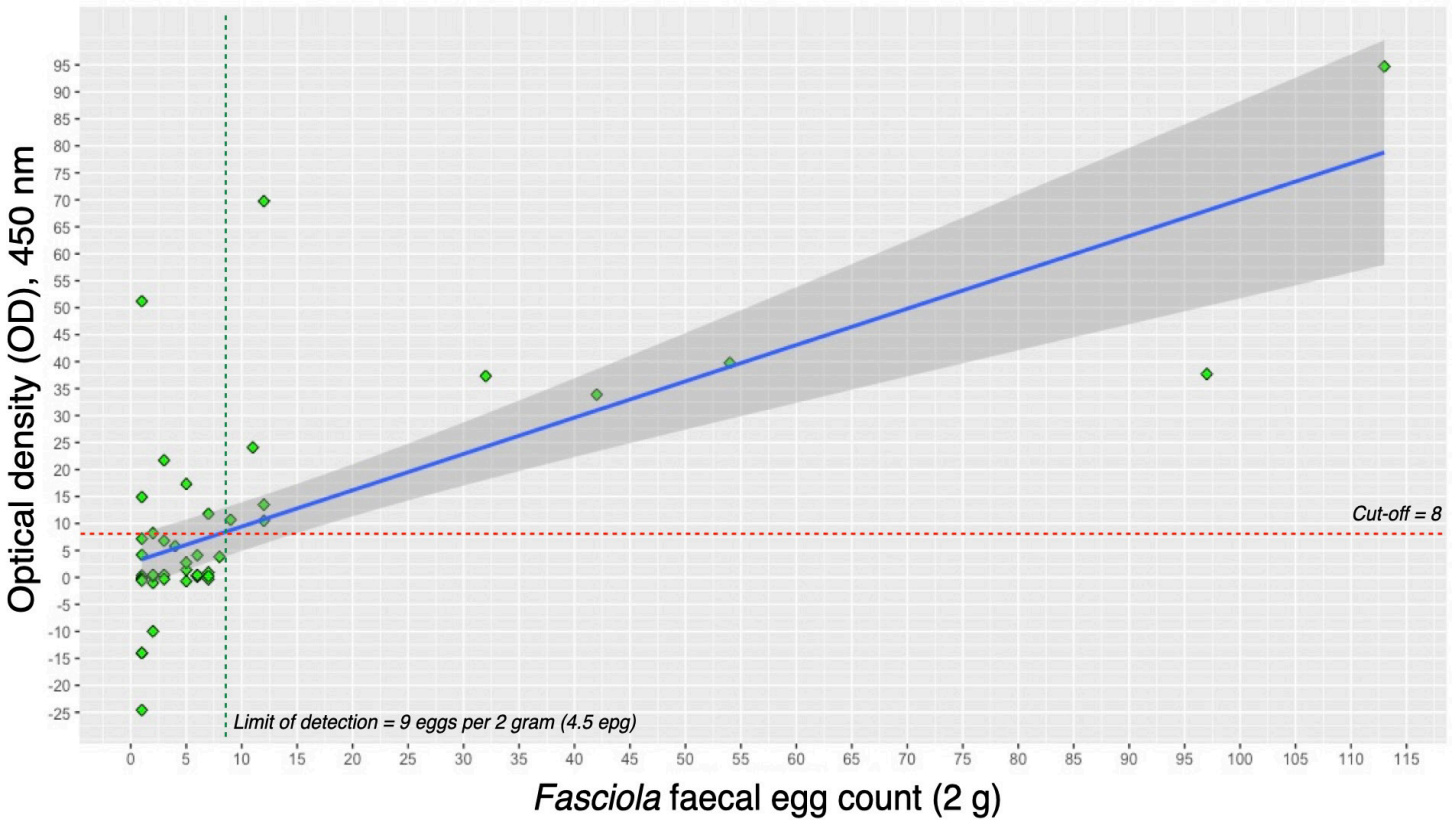
Linear correlation between *Fasciola* faecal egg count (FFEC) and optical density (OD) of coproantigen ELISA (cELISA) for the FFEC+ve group. The correlation coefficient was moderately significant (Spearman’s *r* = 0.716, *p* < 0.01). The limit of detection for 100% positivity in cELISA was 4.5 eggs per gram (epg). The dashed red line indicates the cut-off value (cut-off = 8).

Fig. 1 shows a significant moderate positive correlation between FFEC and *Fasciola* coproantigen concentration as measured by OD readings in the cELISA (Spearman’s *r* = 0.716, *p* < 0.01). Higher egg counts were associated with higher OD values. The cELISA had a lower limit of detection (LoD) of approximately 4.5 epg in the FFEC+ve group, with samples above this threshold showing a 100% positivity rate. The highest OD value corresponded to the highest egg count of 113 eggs per 2 grams (56.5 epg).

Tables 1 and 2 present the descriptive overview of the Flukefinder® and cELISA results. In the FFEC+ve group, 36 out of 46 samples had 4.5 epg or fewer, with only 6 (16.7%) testing positive via cELISA. In contrast, all 10 samples with more than 4.5 epg tested positive. The OD values for cELISA-positive samples ranged from 11 to 94, with a median of 34. In the FFEC-ve group, five samples (10.9%) tested positive for *Fasciola* coproantigen, with OD values ranging from 11 to 39 and a median of 16.

To further investigate the diagnostic performance of the cELISA, the present study stratified FFEC-positive samples by eggs per gram (epg) (Table 2). Sensitivity remained low across all samples with epg values below 4.5, ranging from 0%–25%, but reached 100% at 4.5 epg.

## DISCUSSION

Early detection of fasciolosis in ruminant livestock is essential for effective parasite control and management (Flanagan *et al*. 2011; Martínez-Sernández *et al*. 2016). The present study compared the sensitivity of two diagnostic which were faecal sedimentation by Flukefinder® and a coproantigen ELISA (cELISA) for diagnosing fasciolosis in free-grazing cattle in an area endemic *F. gigantica*. This study is important to enhance the understanding of cELISA’s applicability in monitoring parasite egg counts and preventing the spread of fasciolosis, which can result in continuous environmental contamination with *Fasciola* eggs, especially in free-ranging animals (Tran *et al*. 2022; Che-Kamaruddin *et al*. 2024).

Among the FFEC+ve group, only 16 samples tested positive in cELISA, yielding an overall positivity rate of 34.7%. The present study revealed that samples with more than 4.5 epg showed a 100% positivity rate, while samples with fewer eggs were often cELISA-negative. In field conditions, where free-grazing cattle are exposed to variable parasite challenge levels due to seasonal changes, pasture contamination, and host immunity, cELISA’s lower sensitivity at egg per gram (epg) values below 4.5 poses challenges for its use in screening or surveillance programs. Similar limitations have been reported by previous studies showing inconsistent cELISA positivity in animals with egg counts below 10 epg, particularly in low-challenge settings (George *et al*. 2019). This contrasts with controlled experimental studies, where cELISA reliably detects infections due to higher and more consistent parasite burdens (Mezo *et al*. 2004). The variability in field conditions, including intermittent egg shedding and low antigen levels in early or waning infections, likely contributes to cELISA’s reduced sensitivity compared to Flukefinder®, which detects eggs regardless of fluke viability (Flanagan *et al*. 2011; Kajugu *et al*. 2015; Kelley *et al*. 2021). The discrepancy between cELISA and Flukefinder® highlights a key diagnostic limitation, where the detection of a small number of eggs does not necessarily correspond to an active, high-burden infection producing sufficient coproantigen levels for cELISA detection. Eggs may be detectable in faeces even after fluke clearance, particularly post-treatment, leading to false positives in coproscopic examinations like Flukefinder® (Flanagan *et al*. 2011; Kelley *et al*. 2021). Conversely, cELISA with a detection limit of approximately 4.5 epg, is more sensitive for detecting active infections associated with metabolically active juvenile or adult fluke (Mezo *et al*. 2004; Valero *et al*. 2009; Kajugu *et al*. 2015).

The variability in egg counts among naturally infected animals, often highly skewed (Flanagan *et al*. 2011; Morgan *et al*. 2019; Che-Kamaruddin & Isa 2023), may further explain the limitations of cELISA in detecting low-level infections. This study corroborates that cELISA, while sensitive for detecting active *Fasciola* infections, shows reduced sensitivity at low infection intensities, especially in animals under low challenge conditions (George *et al*. 2019). For instance, infected animals with less than 10 epg showed inconsistent cELISA positivity (George *et al*. 2019). At very low egg count, infections may represent either early or waning stages of infection or residual egg shedding unrelated to viable flukes, conditions under which coproantigen levels may remain below cELISA’s threshold. This variability, influenced by factors such as infection stage, host immune response, and individual susceptibility (Flanagan *et al*. 2011; Morgan *et al*. 2019), highlight the complexity of diagnosing parasitic infections in field settings. For instance, cELISA’s sensitivity improves in high-prevalence endemic areas with moderate to heavy infections, whereas in low-prevalence or low-burden settings (Valero *et al*. 2009), such as those encountered in our study, where the cELISA performance declines. This suggests that cELISA is better suited for targeted diagnostic use in known high-risk areas rather than broad surveillance in regions with sporadic or low-intensity infections. In contrast, coproscopical methods like Flukefinder® maintain higher sensitivity for detecting low egg counts, making them more suitable for initial screening in field conditions, despite their inability to confirm active infection (Gordon *et al*. 2012). Consequently, integrating cELISA with coproscopical or molecular assays could enhance diagnostic accuracy in field-based surveillance, particularly in low-burden settings where early detection is critical to prevent environmental contamination.

The moderate positive correlation observed between cELISA and egg counts supports reliability of cELISA in predicting *Fasciola* burden through egg counts This finding aligns with previous research findings reporting a positive correlation between coproantigen concentration and *Fasciola* burden in experimentally infected cattle, as confirmed through liver examination (Mezo *et al*. 2004). Furthermore, elevated *Fasciola* coproantigen levels is also correlates with egg counts in naturally infected animals (Gordon *et al*. 2012). The odds ratio of 1.96 (CI: 1.61 ± 2.38, *p* < 0.01) indicates that for each additional egg detected in FFEC, the odds of observing a higher coproantigen concentration (OD reading) in the cELISA nearly doubled. This demonstrates that increasing parasite burden is strongly associated with elevated coproantigen levels, reflecting the active metabolic activity of *Fasciola* within the host. Therefore, this underscores cELISA’s potential as a sensitive tool for estimating *Fasciola* egg counts in field settings.

## CONCLUSIONS

This study demonstrates a moderate positive correlation between *Fasciola* coproantigen concentration and faecal egg count (*p* < 0.01), highlighting cELISA as a promising diagnostic tool for cattle fasciolosis under field condition. With a detection limit of 4.5 eggs per gram achieving 100% positivity, cELISA reliably identifies cattle with significant *Fasciola* egg burdens, supporting targeted treatment strategies to control and prevent fasciolosis. However, its lower sensitivity for low-intensity infections below this threshold compared to faecal egg count methods like Flukefinder® indicates that cELISA is less effective for detecting early or residual infections. Combining cELISA with other diagnostic approaches is recommended to improve detection accuracy in low-burden settings, enhancing overall *Fasciola* management and reducing environmental contamination.

## Figures and Tables

**TABLE 1:** Groups of faecal samples categorised by Flukefinder® results for comparative analysis of *Fasciola* faecal egg count (FFEC) and *Fasciola* coproantigen antigen as measured by cELISA. The table presents the odds ratio (OR) of cELISA positivity relative to FFEC.

Group (based on Flukefinder®)	Frequency of positivity from Flukefinder® (%)	Frequency of positivity from cELISA (%)	Odd ratio of positivity in cELISA
Total faecal samples (*N* = 92)	46 (50.0%)	21 (22.8%)	1.96 (*p*-value < 0.01)
FFEC+ve (*n* = 46)	46 (100.0%)	16 (34.8%)	-
• ≤ 4.5 epg (Range: 0.5–4.5 epg)	36 (100.0%)	6 (16.7%)	*-*
• > 4.5 epg (Range: 5.5–56.5 epg)	10 (100.0%)	10 (100.0%)	*-*
FFEC-ve (*n* = 46)	0 (0.0%)	5 (10.9%)	-

**TABLE 2:** Diagnostic performance of cELISA stratified by *Fasciola* eggs per gram (EPG) in naturally infected cattle under field conditions.

*Fasciola* egg per gram (epg)	Total sample count	Total positive sample	Percentage (%)
0.5	11	2	18.18
1	6	1	16.67
1.5	5	1	20.00
2	1	0	-
2.5	4	1	25.00
3	4	0	-
3.5	4	1	25.00
4	1	0	-
4.5	10	10	100.00

Total overall	46	16	34.78

## Data Availability

The raw datasets supporting this article’s findings are available from Naim Che-Kamaruddin upon written request. Please send a request to naimck@um.edu.my or naimchekamaruddin@gmail.com to request access to the data.
